# Differential Effects of Glutelin-to-Globulin Ratios on the Retrogradation Behavior of Rice Starch

**DOI:** 10.3390/foods15213808

**Published:** 2026-10-23

**Authors:** Chunsen Wu, Guifeng Zha, Xin Gong, Zhen Lu, Benxi Wei, Lunan Guo

**Affiliations:** 1 School of Food Science and Engineering, Yangzhou University, Yangzhou 225127, China; 2 School of Food and Biological Engineering, Jiangsu University, Zhenjiang 212013, China

**Keywords:** rice starch gel, glutelin, globulin, anti-retrogradation, protein–starch interaction

## Abstract

Glutelin and globulin in protein body II (PB-II) are the major components of rice protein. This work systematically investigated how varying glutelin-to-globulin ratios (1:0, 2:1, 1:1, 1:2, 0:1) influenced rice starch retrogradation. Glutelin showed strong anti-retrogradation performance, achieving a 54.0% reduction in retrogradation enthalpy (ΔH) and 27.3% inhibition of crystallinity, likely owing to its hydrogen-bonding capacity and network-forming properties. In comparison, globulin increased moisture retention, possibly through competitive hydration, but showed only 7.6% inhibition of crystallinity. Notably, based on ΔH, the 1:1 (w/w) glutelin–globulin blend achieved 83.0% retrogradation inhibition efficiency, comparable to glutelin alone, despite containing half the glutelin content. This phenomenon may result from their different functional roles: glutelin may serve as a structural scaffold, while globulin may act as a water-modulating agent. Overall, glutelin shows superior anti-retrogradation performance to globulin, and the glutelin-to-globulin ratio strongly influences this anti-retrogradation behavior. These results suggest that modulating protein composition may potentially improve storage quality and extend the shelf life of rice starch products.

## 1. Introduction

Rice (*Oryza sativa* L.) is a staple food for more than half the global population and dominates Asian agriculture, with the region accounting for 90% of worldwide production [1]. Although rice is processed into value-added products like instant rice, rice cake, rice gel, and gluten-free snacks, these products experience quality deterioration from starch retrogradation during storage, leading to textural hardening, shortened shelf life, and food waste [2,3]. Therefore, addressing this challenge requires targeted strategies to control retrogradation during modern rice processing.

Emerging evidence shows that rice proteins, known for their hypoallergenic nature, digestibility, and nutritional benefits, effectively modulate starch gelatinization and retrogradation [4,5,6,7]. Rice proteins modulate pasting behavior through water binding and disulphide cross-linked protein networks [4], and delay long-term retrogradation via steric hindrance and restricted water migration [8]. The modulation is dose-dependent: incorporation of 0–10% rice protein inhibits starch cross-linking and delays long-term retrogradation, whereas higher protein concentrations reduce this inhibitory effect [9]. In waxy rice starch, rice protein modulates retrogradation through competitive water-binding and physical-barrier effects, lowering retrogradation enthalpy, crystallinity, and gel hardness while maintaining a porous gel network [10]. Rice protein hydrolysates also suppress short- and long-term retrogradation in wheat starch systems [11], suggesting broader food applications. Although anti-retrogradation effects depend on starch and protein type, starch-protein ratio, and processing/storage conditions [12], the underlying mechanisms remain poorly understood.

Rice protein comprises four major fractions: glutelin, globulin, prolamin, and albumin. Glutelin and globulin, the two principal components of rice protein body II (PB-II), possess fundamentally distinct molecular characteristics [13,14]. Glutelin is a flexible polypeptide chain rich in glutamine, asparagine, and hydroxyl-containing residues, which results in a high density of hydrogen-bonding donors and acceptors [15,16,17]. In contrast, globulin is a salt-soluble globular protein characterized by a compact tertiary structure, a higher proportion of ionizable amino acids, and a strong capacity for ionic hydration [18]. These molecular features lead to distinct interactions with starch. Glutelin increases pasting temperature and enhances gel adhesiveness while suppressing peak, breakdown, and final viscosities, primarily by forming viscoelastic networks that restrict water movement and stabilize starch granules [18,19,20,21]. Globulin, in contrast, slightly lowers pasting temperature, linearly reduces all viscosity parameters, and decreases adhesiveness, but increases gel hardness—although its specific influence can vary with dose and source, as observed in soybean and wheat systems [18,22,23]. When both glutelin and globulin are added to rice starch, their effects on viscosity and gel hardness are additive, whereas their effects on pasting temperature and adhesiveness are antagonistic and depend on their relative concentrations [18]. A comparable ratio-dependent regulation is well documented in wheat gluten, where the gliadin/glutenin ratio critically influences starch gelatinization behavior, with gliadin reducing peak viscosity more than glutenin and increasing enthalpy through distinct hydrophobic and hydrogen bonding interactions that alter starch molecular order [24]. Adjusting this ratio in reconstituted gluten fractions can modify granule swelling, disruption, and amylose leaching, thereby tuning rheological properties and gel strength while inhibiting retrogradation [25]. These findings suggest that the glutelin/globulin ratio in rice protein may similarly regulate starch structural organization and retrogradation. Beyond glutelin and globulin, other fractions also contribute: prolamin has been found to increase breakdown viscosity and soften starch gels, further highlighting divergent functions among rice protein fractions [26]. Despite these findings, how glutelin and globulin, individually and in combination, modulate starch structural organization and retrogradation, and how their ratio governs this process, remains poorly understood.

Based on these findings, this study systematically investigates the effects of varying glutelin-to-globulin ratios (glu/glo = 1:0, 2:1, 1:1, 1:2, 0:1) on rice starch retrogradation by comprehensively evaluating structural, textural, thermal, moisture retention, water mobility, and morphological properties. These findings will provide actionable strategies for developing stable rice-based foods with controlled retrogradation and extended shelf life.

## 2. Materials and Methods

### 2.1. Materials

*Japonica* rice (*Oryza sativa* L. *japonica*) was procured from China Oil and Foodstuffs Corporation (COFCO, Beijing, China). Petroleum ether, sodium chloride, and sodium hydroxide were purchased from Sinopharm Chemical Reagent Corporation Limited (Shanghai, China). All other chemicals were of analytical grade and required no further purification.

### 2.2. Rice Starch Isolation

Starch was prepared using an alkaline steeping method [27]. Milled rice (200 g) was defatted three times with petroleum ether (1:4 w/v, 30 min agitation per cycle) and centrifuged (4000× *g*, 15 min, 25 °C). The residue was air-dried overnight (24 h, 25 °C), suspended in 0.3% NaOH (1:4 w/v), stirred (250 rpm, 4 h), and centrifuged (4000× *g*, 20 min). After triple processing, the starch layer was neutralized via sequential washing (deionized water, pH 7.0), oven-dried (24 h, 45 °C), sieved (200-mesh), and stored desiccated at 4 °C.

### 2.3. Globulin and Glutelin Extraction

Globulin and glutelin were isolated using a modified Osborne sequential protein extraction method [28]. Briefly, defatted rice flour (50 g) underwent sequential extraction following Osborne classification. The flour was dispersed in distilled water (1:5 w/v) and stirred at 25 °C for 2 h to remove albumin. After centrifugation at 10,000 *g* for 10 min, the supernatant was discarded, and the pellet was retained. The pellet was extracted with 5% NaCl solution (1:5 w/v, 2 h, 25 °C) and centrifuged at 10,000 *g* for 10 min. The supernatant was adjusted to pH 4.3 for isoelectric precipitation to obtain globulin. To isolate glutelin, the residue was treated with 70% ethanol to remove prolamin. After centrifugation, the remaining solid was extracted with 0.02 M NaOH solution (1:5 w/v, 2 h), centrifuged (10,000× *g*, 10 min), and precipitated at pH 4.8. Both proteins were washed three times, dialyzed overnight in distilled water until reaching neutral pH, freeze-dried, sieved (100-mesh), and stored at 4 °C.

### 2.4. Composite Gel Preparation

Starch-protein composite gels were prepared by blending rice starch (88%, w/w) with glutelin–globulin mixtures at mass ratios of 1:0, 2:1, 1:1, 1:2, and 0:1. The pure rice starch gel (PRSG) served as the control. Dry mixtures were combined with deionized water (1:2 w/v), molded, stirred, steamed at 100 °C for 3 min, cooled at 25 °C for 2 h, vacuum-sealed, and stored at 25 °C for 14 d. Following storage, a portion of each gel was freeze-dried for morphological observation and ground to pass through a 200-mesh sieve for structural analysis.

### 2.5. Moisture Content Analysis

Moisture content was determined gravimetrically using modified methods [29]. Gel cores (~2.0 g) were dried (105 °C, 24 h) in pre-weighed aluminum crucibles. Each sample was measured in triplicate as follows:

(1) $$X=\frac{m_{1}-m_{2}}{m_{1}-m_{3}}\times 100\%$$

where X = moisture content (%), m_1_ = initial mass (g), m_2_ = post-drying mass (g), m_3_ = crucible mass (g).

### 2.6. Texture Profile Analysis (TPA)

Gel texture was measured using a TA-XT2 texture analyzer (Stable Micro Systems, Godalming, Surrey, UK) with a TA/75 probe, following published protocols [29,30]. Cylindrical samples (3 cm × 1.5 cm × 1 cm) were subjected to two-cycle compression at 30% strain, 0.1 mm/s, and a 4.0 g trigger force. Hardness, cohesiveness, springiness, and resilience were calculated from force-time curves using Exponent software (v6.1, Stable Micro Systems, Godalming, Surrey, UK).

### 2.7. Differential Scanning Calorimetry (DSC)

Thermal properties were analyzed using a DSC 200 F3 (NETZSCH-Gerätebau, Selb, Germany). The gel-water mixture (3:2 w/w, 10 mg total mass) was hermetically sealed in aluminum pans, equilibrated (4 °C, 24 h), and scanned (25–100 °C, 5 °C/min under N_2_). Onset temperature (T_o_), peak temperature (T_p_), conclusion temperature (T_c_), and enthalpy (ΔH) were recorded.

### 2.8. Fourier Transform Infrared Spectroscopy (FTIR) Measurements

Freeze-dried gels were ground with KBr (1:150 w/w), pelleted (10 MPa, 1 min), and analyzed using FTIR systems (Cary 5000, Varian, Palo Alto, CA, USA) following a modified method [31]. The system was operated in transmittance mode (400–4000 cm−1, 4 cm^−1^ resolution, 16 scans).

### 2.9. X-Ray Diffraction (XRD)

The XRD patterns were analyzed via a D8 Advance XRD (Bruker-AXS, Karlsruhe, Germany) following a modified literature procedure [32]. Briefly, diffractograms were obtained from 4 to 40° with a step size of 3°/min at 20 kV.

### 2.10. Small-Angle X-Ray Scattering (SAXS)

SAXS measurements were conducted on a NanoSTAR instrument (Bruker-AXS, Karlsruhe, Germany) equipped with Cu-Kα radiation (λ = 0.154 nm, 40 kV, 50 mA) using a reported method [33]. Freeze-dried starch gels were suspended in deionized water (1:3 w/v), equilibrated (25 °C, 24 h), centrifuged (8000× *g*, 10 min), and analyzed in transmission mode (0.01–0.2 Å^−1^, 60 s exposure), fractal dimensions (D) were calculated via [34]:

(2) $$d=\frac{2\pi }{q}$$

(3) $$I(q)\sim q^{\alpha }$$

D_m_ = −α (4)

where d is semi-crystalline layer thickness (nm); q is scattering vector (Å^−1^); α is scaling exponent; I(q) is normalized intensity (a.u.); D_m_ is a mass fractal dimension.

### 2.11. Scanning Electron Microscopy (SEM) Observation

Freeze-dried starch gels were sputter-coated and imaged using a Gemini SEM 300 (Carl Zeiss, Oberkochen, Germany) at an extra high tension of 5.0 kV and 1000× magnification.

### 2.12. Low-Field Nuclear Magnetic Resonance (LF-NMR)

Water mobility was analyzed using a PQ001 LF-NMR (Niumag Corporation, Suzhou, China) at designed storage intervals (0, 1, and 14 days) using the previously described procedure [35]. Transverse relaxation time (T_2_) was acquired through Carr-Purcell-Meiboom-Gill pulse sequences with optimized parameters: 1024 sampling points, 8 accumulated scans, and a 1000–2000 ms relaxation decay. Multi-exponential decay curves were deconvoluted using Niumag T_2_ cloud v1.0 software.

### 2.13. Statistical Analysis

All experimental data were obtained from three separately prepared gel batches (*n* = 3) and expressed as mean values ± standard deviation (SD). Statistical comparisons among multiple groups were performed using one-way ANOVA (Duncan’s test) in IBM SPSS Statistics (version 24.0, IBM Corp., Armonk, NY, USA). Between-group differences at *p* < 0.05 were considered statistically significant. Figures were generated using OriginPro (version 2021, OriginLab Corporation, Northampton, MA, USA).

## 3. Results and Discussion

### 3.1. Post-Storage Moisture Content

The purified starch used in this study contained 18.8% amylose, as quantified using the Megazyme amylose/amylopectin assay kit (K-AMYL kit, Megazyme Ltd., Bray, County Wicklow, Ireland). After storage, the glutelin-to-globulin ratios significantly influenced the moisture content of composite rice starch gels (Table 1). Protein-modified gels maintained consistently higher moisture content than the PRSG, with the globulin-only (glu/glo = 0:1) retaining the maximum moisture content (20.39%). The glutelin-only system (glu/glo = 1:0) retained only 68% of the moisture retained by its globulin counterpart, highlighting globulin’s superior water-holding capacity. Moisture retention generally increased with higher globulin content, although the 1:1 (w/w) glutelin–globulin blend showed anomalous behavior. This deviation might be attributed to competing hydration mechanisms: globulin may promote hydration through ionic interactions, whereas glutelin may restrict water accessibility through hydrophobic aggregation [18,36].

### 3.2. Texture Profile Analysis

The textural properties of stored composite rice starch exhibited protein-dependent effects (Table 1). Compared with PRSG, protein-modified gels had significantly lower hardness, gumminess, and chewiness, partially consistent with previous reports on gluten-modified systems [37]. These texture parameters positively correlated with glutelin content but negatively correlated with globulin content. Glutelin may restrict water diffusion within semi-crystalline starch regions, increasing matrix hardness and chewiness through uniform molecular integration with starch molecules [38,39]. Conversely, globulin’s ionic hydration might weaken starch–chain interactions, yielding a softer gel network. Variations in glutelin-to-globulin ratios altered the springiness and cohesiveness of the rice starch gel within a narrow range. The 1:1 (w/w) glutelin–globulin blend exhibited the highest cohesiveness. In reconsulted rice, springiness correlated less with the glutelin-to-rice starch ratio but still benefited the formation of a dense, homogeneous, continuous structure [38]. Modulating glutelin-to-globulin ratios might balance the opposing effects of glutelin and globulin on rice starch texture.

### 3.3. Thermal Properties

DSC was used to quantify the thermodynamic changes associated with the melting of recrystallized amylopectin double helices, providing a direct measure of retrogradation extent. DSC analysis revealed elevated gelatinization temperatures (T_o_, T_p_, and T_c_) in all protein-modified gels (Table 2), likely due to the higher denaturation temperature of rice proteins [40]. PRSG exhibited the highest retrogradation enthalpy (ΔH = 3.91 J/g), whereas protein-modified systems exhibited reduced ΔH values (1.80–2.71 J/g). Glutelin demonstrated superior inhibition efficiency (46.0% ΔH retention vs. PRSG) compared to globulin (69.3% ΔH retention vs. PRSG). In wheat protein studies, glutenin also showed superior retrogradation inhibition to gliadin [25]. The anti-retrogradation effect of glutelin might be attributed to its ability to form hydrogen bonds with starch molecules, stabilizing the internal structure [38]. Notably, the 1:1 (w/w) glutelin–globulin blend achieved retrogradation inhibition (55.2% ΔH retention vs. PRSG) comparable to that of the glutelin-only system (46.0% ΔH retention vs. PRSG), despite containing half the glutelin content. 

### 3.4. FTIR Analysis

**Figure 1:**
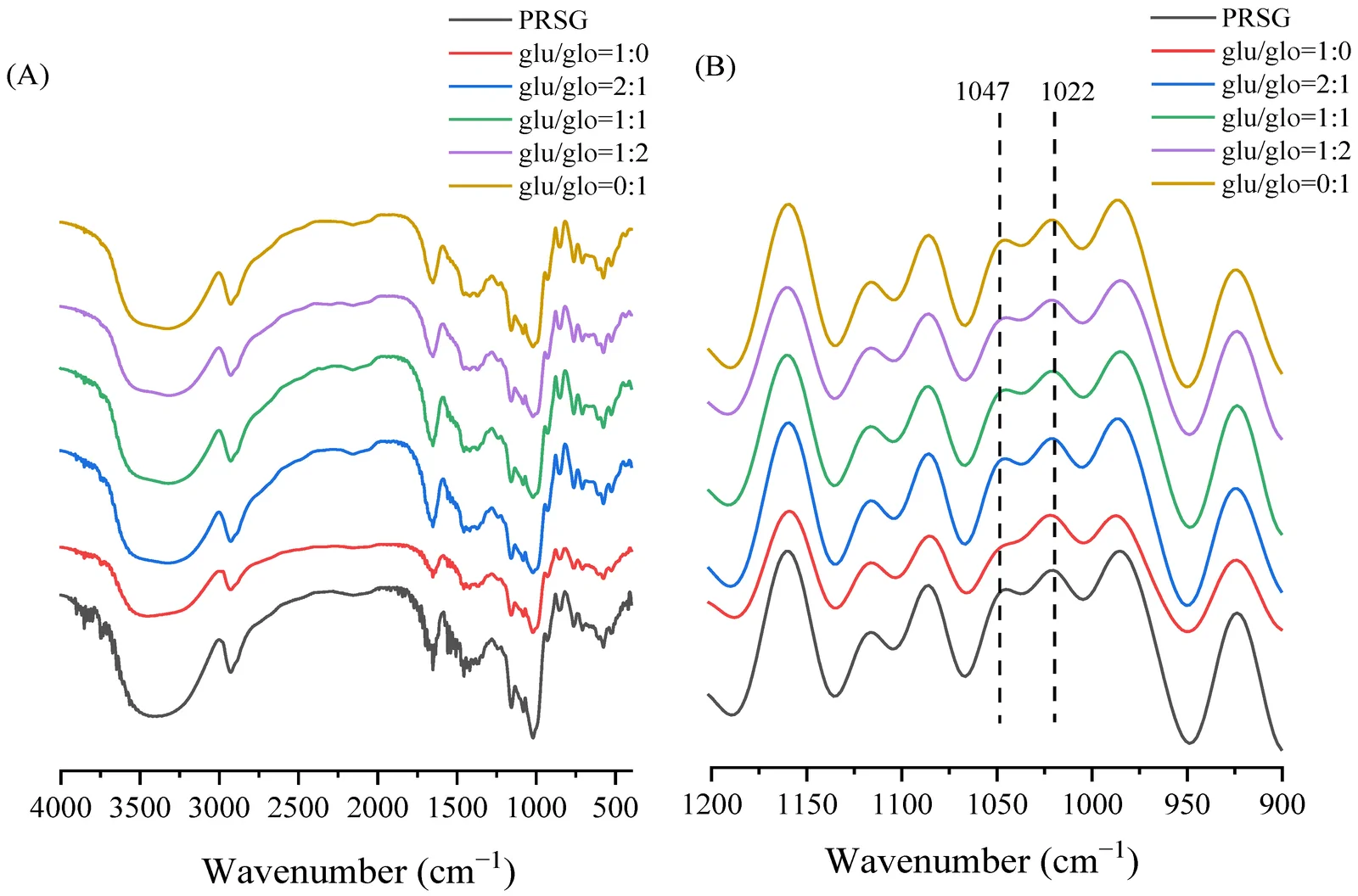
FTIR spectroscopy (**A**) and deconvolution spectra (**B**) of rice gels modified by different glutelin-to-globulin ratios after 14 days storage.

FTIR spectroscopy was used to assess short-range molecular order and hydrogen bonding interactions between starch and proteins, as indicated by the 1047/1022 absorbance ratio. FTIR spectra and spectral deconvolution revealed alterations in molecular interactions within stored starch gels (Figure 1). All samples exhibited characteristic absorption bands in the 3600–3000 cm^−1^ region, corresponding to O-H stretching vibrations of starch hydroxyl groups engaged in hydrogen-bonding networks [35]. PRSG showed the broadest absorption peak in this region, indicating the most extensive hydrogen-bonded networks. In contrast, the glutelin-only system (glu/glo = 1:0) showed pronounced peak narrowing, consistent with disruption of starch hydroxyl interactions, which may be attributable to the extended polypeptide chains of glutelin [17,18]. The 1047/1022 cm^−1^ absorbance ratio, which indicates crystalline-to-amorphous transitions in starch granules [41], varied significantly across the modified systems (Table 2). Protein-modified gels showed significantly lower ratios than PRSG (0.8114), with glutelin causing the greatest reduction. Interestingly, the 1:1 (w/w) glutelin–globulin blend achieved a ratio reduction comparable to the glutelin-only system.

### 3.5. XRD Analysis

**Figure 2:**
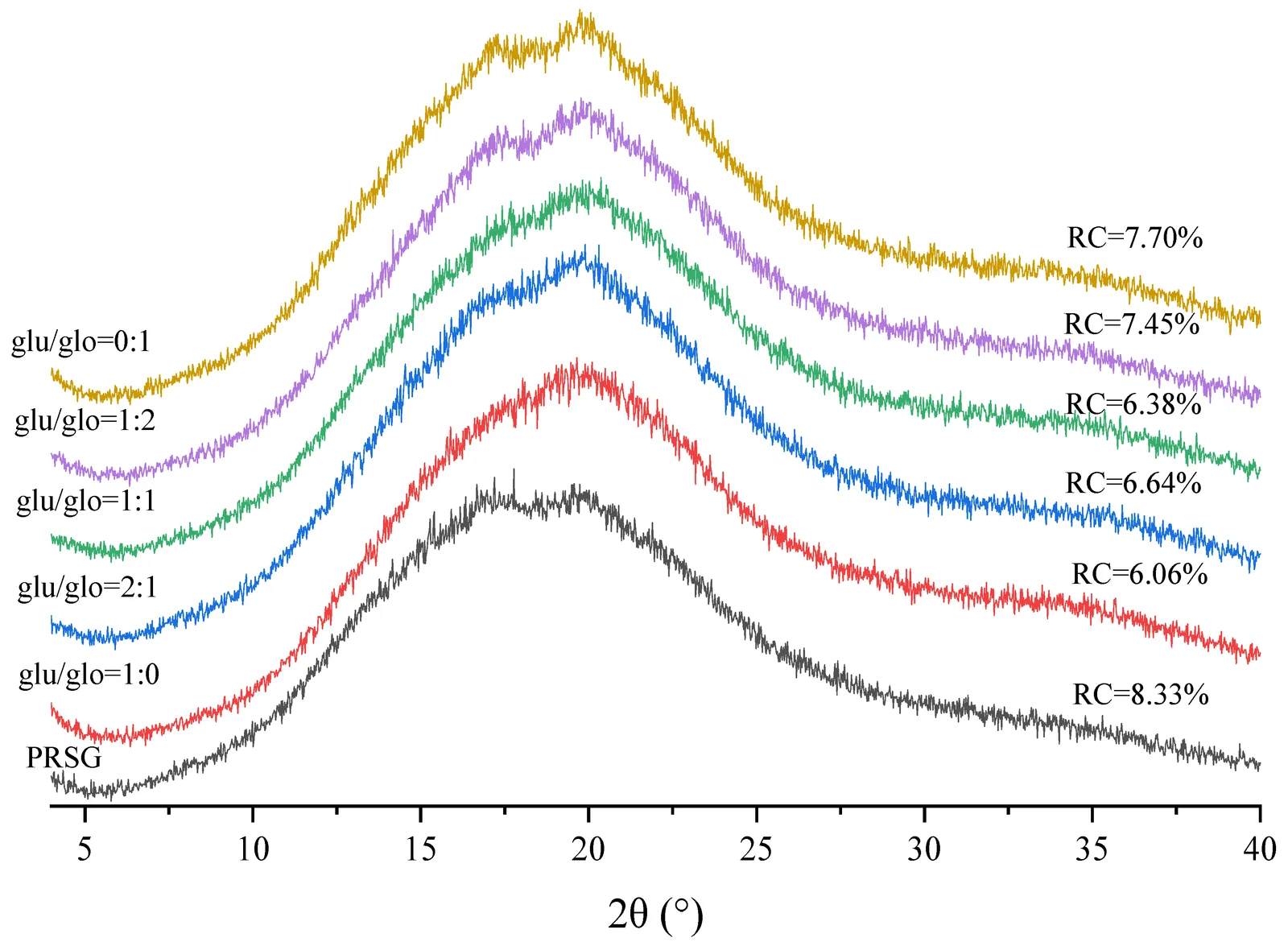
XRD pattern and crystallinity of rice starch gels modified by different glutelin-to-globulin ratios after 14 days storage.

XRD analysis was performed to assess the long-range crystalline order and crystal type of retrograded starch, with the relative crystallinity as an indicator of recrystallization. As illustrated in Figure 2, XRD profiles confirmed B-type crystalline patterns (2*θ* ≈ 17°) in all stored rice starch gels, indicating protein incorporation did not alter starch polymorphism. PRSG exhibited 8.33% relative crystallinity, while all protein-modified gels showed lower values, consistent with previous studies on gluten-, porcine plasma protein-, and whey protein-starch systems [25,41,42]. Glutelin (glu/glo = 1:0) achieved 27.3% crystallinity inhibition, outperforming globulin (glu/glo = 0:1) with 7.6% inhibition. This non-monotonic trend highlights glutelin’s superior anti-retrogradation ability, likely due to polypeptide entanglement and noncovalent bonding in the amorphous region [25]. Notably, the 1:1 (w/w) glutelin–globulin blend unexpectedly matched pure glutelin in crystallinity inhibition, consistent with the FTIR observations.

### 3.6. SAXS Analysis

**Figure 3:**
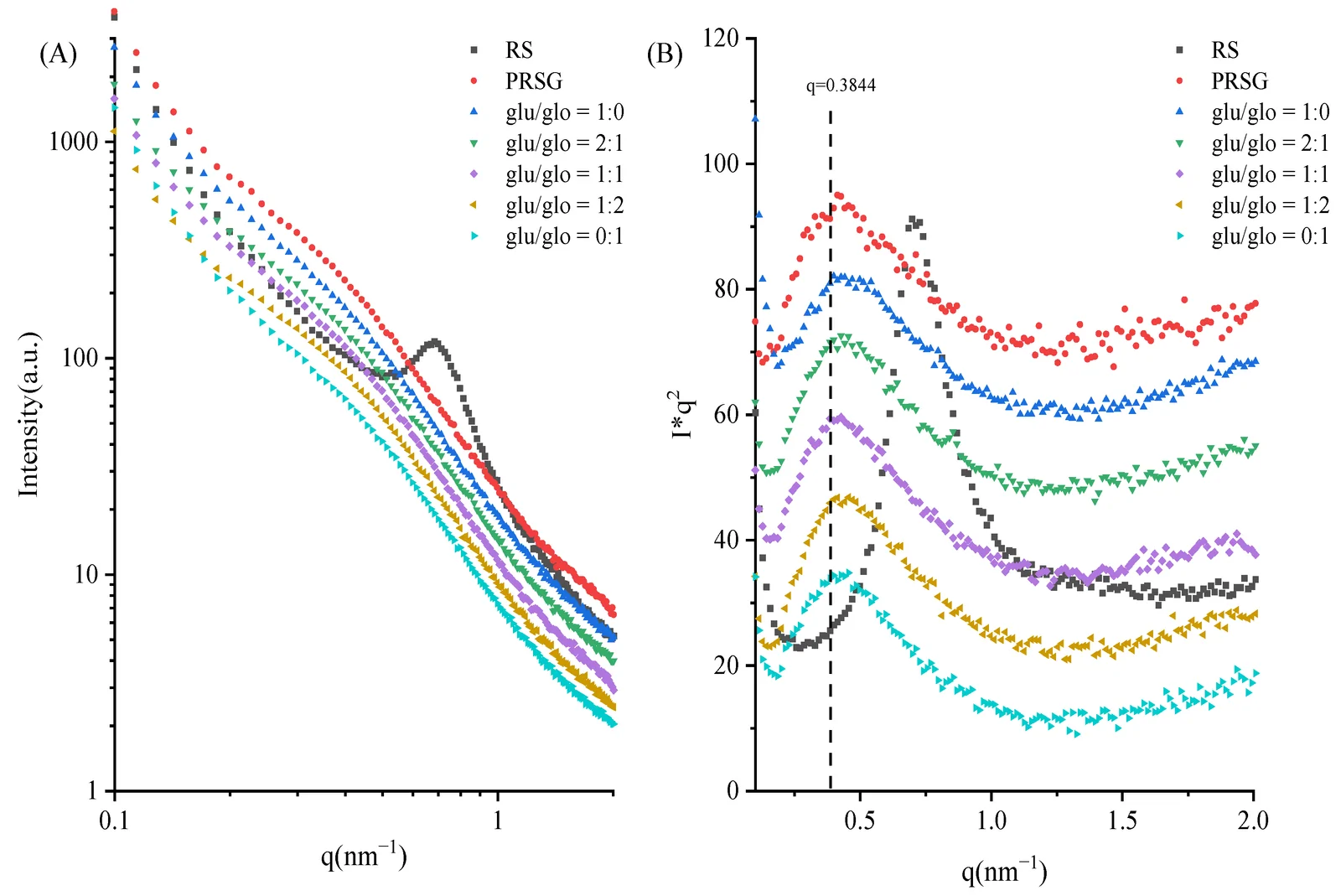
SAXS patterns (**A**) and Lorentz transformation patterns (**B**) of rice starch gels modified by different glutelin-to-globulin ratios after 14 days storage.

SAXS was employed to characterize the nanoscale lamellar architecture, including lamellar spacing and fractal dimension, of the retrograded starch gels [43]. Figure 3A reveals a characteristic shoulder-like peak at low *q*-values for all gels after storage, corresponding to the periodic arrangement of crystalline and amorphous lamellae during amylopectin recrystallization [44]. To enhance the visibility of the shoulder-like peaks, Lorentz transformation analysis (Figure 3B) revealed a distinct peak at q = 0.3844 nm^−1^ for PRSG and higher values in protein-modified gels, indicating reduced lamellar spacing in the semi-crystalline lamellar structure. The diminished peak area (Table 2) in protein-modified gels, especially for glu/glo = 1:0, further supports the suppression of semi-crystalline domain formation, consistent with XRD and FTIR results. This phenomenon indicates that protein-starch entanglement disrupts the regular alternation of crystalline and amorphous regions [43,44,45]. Furthermore, both α and D_m_ values decreased with glutelin and globulin incorporation, indicating reduced starch structural compactness. These results indicate that glutelin exerted the most pronounced effect, disrupting the semi-crystalline lamellar architecture.

### 3.7. Microstructural Characterization

**Figure 4:**
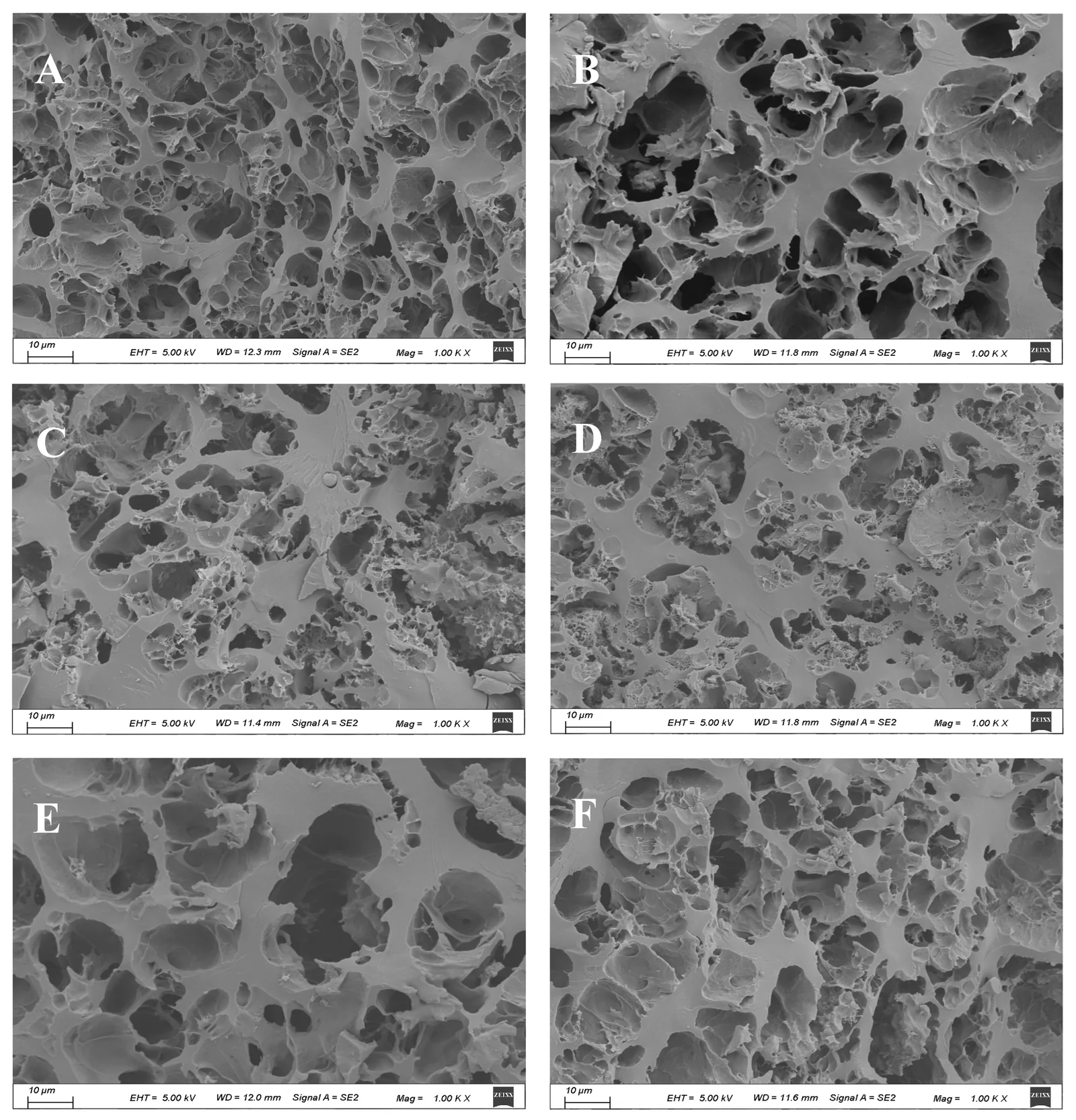
Scanning electron microscopy (SEM) images of rice starch gels modified by different glutelin-to-globulin ratios after 14 days storage; (**A**–**F**) in turn represent PRSG, glu/glo = 1:0, glu/glo = 2:1, glu/glo = 1:1, glu/glo = 1:2, and glu/glo = 0:1.

SEM revealed distinct protein-dependent microstructural patterns in lyophilized stored starch gels (Figure 4). PRSG showed a heterogeneous porous structure with fractured thin matrix walls, characteristic of extensive retrogradation-driven phase separation and syneresis [9]. The glutelin-modified system (glu/glo = 1:0) formed a continuous, reinforced network via glutelin-starch entanglement, stabilizing the matrix, whereas the globulin-dominant system (glu/glo = 0:1) showed partial collapse with anisotropic pores. Glutelin exhibits superior gap-filling ability because glutelin likely forms hydrogen-bonded networks that resist collapse, whereas globulin’s water competition limits starch-chain mobility. The 1:1 (w/w) glutelin–globulin blend formed an intermediate architecture, more consolidated than the globulin-only gels yet less dense than the glutelin-only gels, suggesting that glutelin’s network-forming capacity and globulin’s hydration effects may act together to preserve structural integrity [18,46].

### 3.8. LF-NMR Analysis

**Figure 5:**
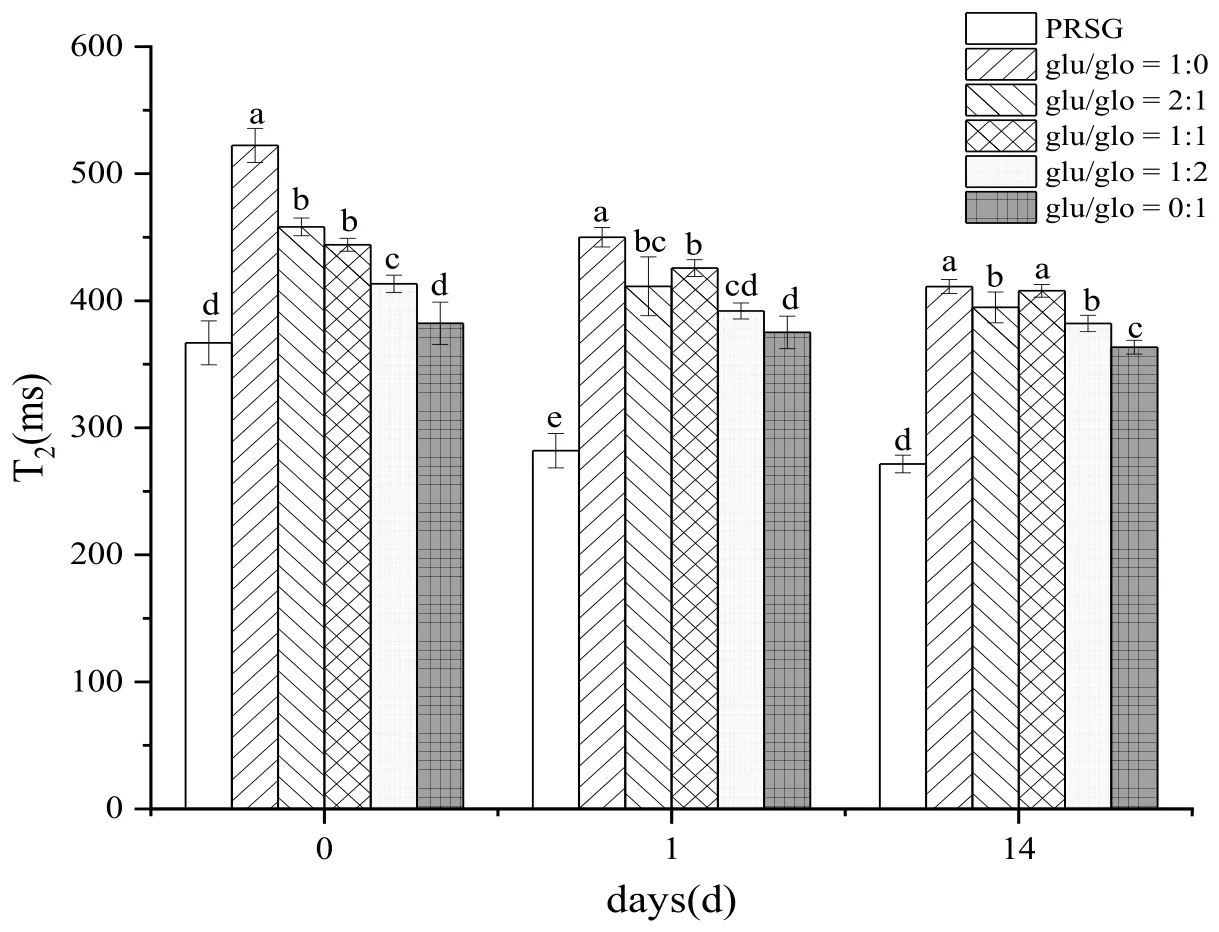
Changes in relaxation time (T2) of rice starch gels modified by different glutelin-to-globulin ratios during 14 days storage. Different lowercase letters within each storage duration indicate significant differences between values (*p* < 0.05).

LF-NMR was utilized to monitor water distribution and mobility (T_2_ relaxation times) within the gel matrix [47]. The spin-spin relaxation time (T_2_) is directly proportional to water mobility; higher T_2_ values indicate greater water mobility [8]. In Figure 5, T_2_ values for all samples decreased during storage, indicating reduced water mobility [48]. Protein-modified systems systematically increased T_2_ values, with the glutelin-only incorporation showing the most pronounced effect, suggesting that glutelin promoted water mobility more effectively than globulin [18,19,20,21]. Globulin may act as a water binder in the food matrix, likely because of its superior water-holding capacity, competing with starch granules for available water [18,22,23]. Interestingly, the 1:1 (w/w) glutelin–globulin blend exhibited a T_2_ value slightly lower than that of pure glutelin (*p* > 0.05), indicating that adjusting the glutelin-to-globulin ratio might modulate water mobility and consequently influence the anti-retrogradation effect.

**Figure 6:**
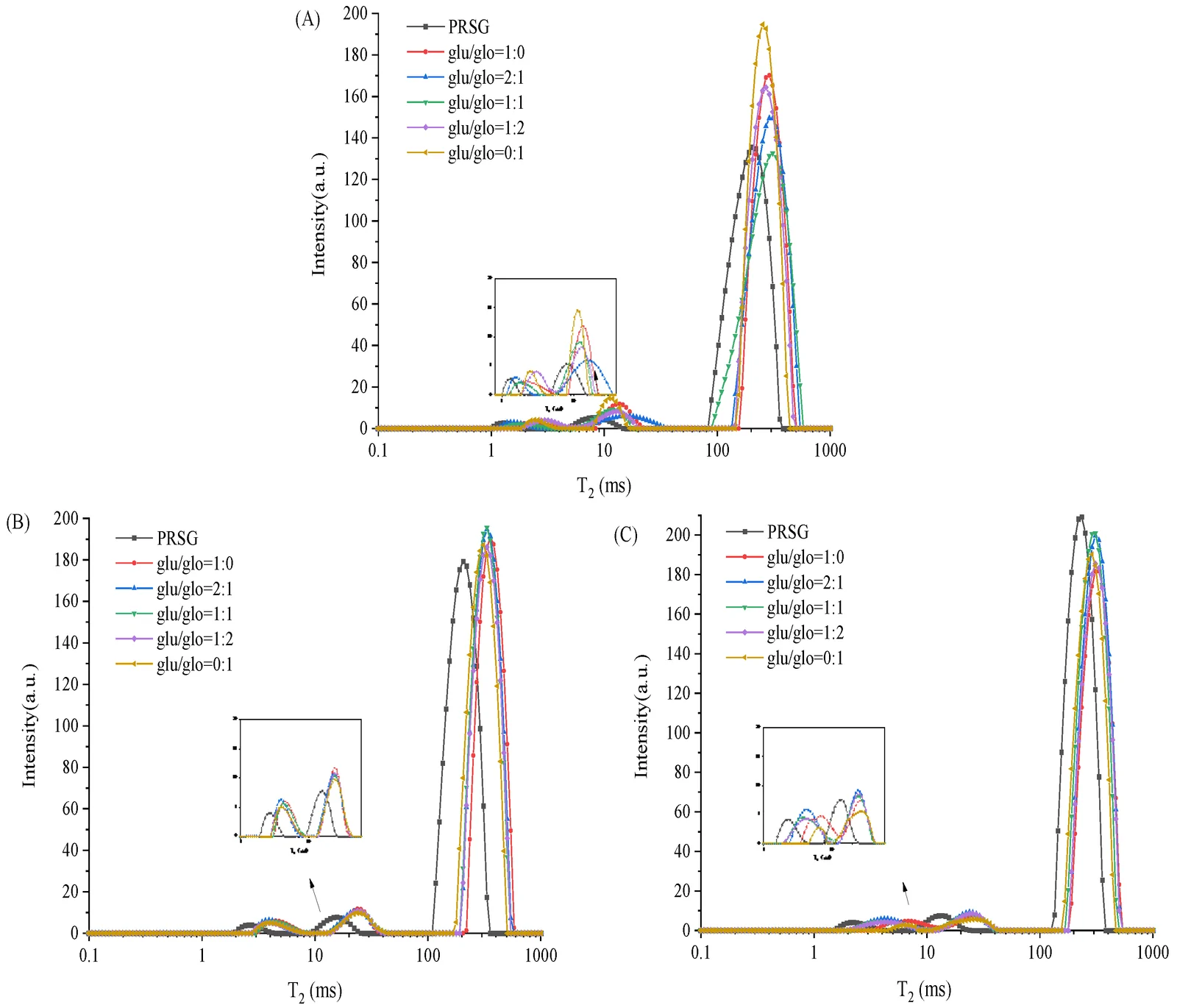
Distribution of relaxation time (T2) of rice starch gels modified by different glutelin-to-globulin ratios during 14 days storage. 0 d (**A**); 1 d (**B**); 14 d (**C**). The black arrow indicated the region shown at higher magnification in the inset figure.

In Figure 6, all systems exhibited tri-exponential decay patterns with three distinct water populations: tightly bound (T_21_:1–10 ms), weakly bound (T_22_:10–100 ms), and free water (T_23_:100–1000 ms) [8]. Compared to PRSG, T_21_, T_22_, and T_23_ for protein-modified samples all showed noticeable prolongation, with the glutelin system (glu/glo = 1:0) showing the highest values, suggesting the strongest water mobility [8,46]. Moreover, all protein-modified samples reduced the T_23_ peak area, indicating that glutelin and globulin retain water molecules that do not readily transform into free water during storage, thereby inhibiting retrogradation in rice starch gels [8,46].

### 3.9. Integrated Discussion of Anti-Retrogradation Mechanisms

Multiple analytical methods were used to assess the anti-retrogradation effects of glutelin, globulin, and their blends. DSC results indicated that glutelin achieved the greatest reduction in retrogradation enthalpy (46.0% ΔH retention vs. PRSG). FTIR analysis showed a narrower O-H stretching at 3600–3000 cm^−1^, suggesting hydrogen bonding between glutelin and starch hydroxyl groups [35]. These interactions may disrupt starch molecular ordering, thereby reducing lamellar spacing and lowering relative crystallinity by 27.3%. SEM images showed glutelin-modified starch formed continuous, denser matrices, likely restricting starch chain rearrangement. Similar steric hindrance-based anti-retrogradation effects have been observed in hydrolyzed whey protein starch systems [41].

In comparison, globulin exhibited weaker anti-retrogradation effects (69.3% ΔH retention vs. PRSG) and only 7.6% crystallinity inhibition. However, globulin exhibited stronger water-holding capacity (20.39%). Prolonged water relaxation times in LF-NMR further indicated altered water distribution in globulin-containing systems. Prolamin, another rice storage protein, has also been reported to affect the water absorption behavior in rice starch during cooking [26]. The globulin is proposed to compete with starch for water molecules through ionic hydration, partially limiting starch chain mobility [8]. A similar water-competition phenomenon occurs in waxy rice starch gels, where rice protein restricts water mobility and disrupts starch chain cross-linking, thereby inhibiting retrogradation [10]. These properties suggest globulin may suit applications requiring high moisture retention, such as refrigerated and frozen starch products that need a soft texture rather than long-term storage stability [49,50].

The 1:1 (w/w) glutelin–globulin blend achieved desirable overall performance by integrating the advantages of both proteins. Its retrogradation enthalpy reduction (55.2% ΔH retention vs. PRSG) was comparable to glutelin alone, while FTIR revealed a distinct hydrogen-bonding pattern for this co-blended system. Texture analysis also showed maximal cohesiveness and moderate hardness, meeting general food processing requirements [12,37]. Based on retrogradation enthalpy (ΔH), the 1:1 (w/w) glutelin–globulin blend achieved about 83.0% retrogradation inhibition efficiency of glutelin alone, despite containing half the glutelin content. This response might be conceptualized as follows: glutelin may provide a structural scaffold that directly impedes starch chain re-association, while globulin might act as a water-modulating agent. At this blending ratio, the glutelin network may retain mechanical integrity, consistent with the thickened network walls observed in SEM images and moderate hardness, and globulin’s hydration competition may create an environment unfavorable for crystal growth. In the literature, wheat glutenin and gliadin play different roles in regulating starch retrogradation: glutenin inhibits long-term retrogradation through disulfide-linked networks, whereas gliadin suppresses short-term retrogradation by competing for free water in the system [24,25]. Reasonable regulation of the ratio of these two wheat proteins enables targeted modulation of starch retrogradation, effectively improving the shelf stability of wheat-based food products [24,25]. The 1:1 glutelin–globulin blend in the rice starch system achieves a balanced and efficient anti-retrogradation effect by coupling glutelin’s structural barrier function with glutelin’s water-regulating function.

## 4. Conclusions

This study systematically compared the anti-retrogradation behaviors of rice glutelin, globulin, and their blends in rice starch systems. Compared with PRSG, glutelin showed stronger anti-retrogradation performance (54.0% ΔH reduction and 72.7% residual crystallinity), likely due to stronger hydrogen bonding and network formation. Globulin may improve moisture retention through competitive hydration but interacted weakly with starch, resulting in only 7.6% inhibition of crystallinity. Glutelin exhibited a greater anti-retrogradation capacity than globulin. The glutelin-to-globulin ratios strongly affected the anti-retrogradation behavior. This may be hypothesized to result from their different functional roles: glutelin may serve as a structural scaffold for retrogradation inhibition, while globulin may act as a water-modulating agent for moisture retention. Based on ΔH, the 1:1 (w/w) glutelin–globulin blend achieved an anti-retrogradation inhibition capacity equivalent to 83.0% of pure glutelin, despite containing half the glutelin. These results suggest that modulating glutelin-to-globulin ratios has the potential to maintain product quality and inhibit retrogradation in rice starch-based products during storage. Future work will investigate the molecular interaction mechanism of rice proteins in complex food matrices.

## Figures and Tables

**Table 1:** The moisture content and texture parameters of rice starch gels modified by different glutelin-to-globulin ratios after 14 days storage.

Sample	Moisture Content (%)	Hardness (gf)	Gumminess (gf)	Springiness	Chewiness (gf)	Cohesiveness
PRSG ^1^	12.48 ± 0.39 ^d,2^	758.30 ± 18.03 ^a^	636.27 ± 26.10 ^a^	0.93 ± 0.05 ^a^	589.38 ± 23.99 ^a^	0.84 ± 0.05 ^c^
glu/glo = 1:0 ^3^	13.84 ± 0.75 ^c^	629.06 ± 20.25 ^b^	546.70 ± 18.40 ^b^	0.94 ± 0.06 ^a^	512.07 ± 19.37 ^b^	0.87 ± 0.02 ^bc^
glu/glo = 2:1	16.59 ± 0.62 ^b^	557.97 ± 14.53 ^c^	506.27 ± 17.65 ^c^	0.86 ± 0.04 ^b^	435.66 ± 8.45 ^c^	0.91 ± 0.01 ^ab^
glu/glo = 1:1	16.25 ± 0.49 ^b^	498.14 ± 6.48 ^d^	472.32 ± 12.43 ^d^	0.81 ± 0.03 ^b^	383.94 ± 9.94 ^d^	0.95 ± 0.03 ^bc^
glu/glo = 1:2	19.19 ± 0.57 ^a^	452.61 ± 16.31 ^e^	399.68 ± 28.03 ^e^	0.85 ± 0.05 ^b^	337.74 ± 8.82 ^e^	0.88 ± 0.04 ^bc^
glu/glo = 0:1	20.39 ± 0.25 ^a^	406.20 ± 10.83 ^f^	364.05 ± 24.86 ^f^	0.88 ± 0.02 ^ab^	319.84 ± 19.58 ^e^	0.90 ± 0.04 ^ab^

^1^ PRSG is the pure rice starch gel as a control. ^2^ Values followed by different letters within a column denote significantly different levels (*p* <0.05). ^3^ glu/glo is the glutelin-to-globulin ratio.

**Table 2:** The 1047/1022, SAXS parameters, and thermal properties of rice starch gels modified by different glutelin-to-globulin ratios after 14 days storage.

Sample	FTIR	SAXS					DSC			
	1047/1022 (cm^−1^)	Peak Area	α	D_m_	d (nm)	q (nm^−1^)	T_o_ (°C)	T_p_ (°C)	T_c_ (°C)	∆H (J/g)
PRSG ^1^	0.8114 ± 0.0058 ^a,2^	10.44	−2.36	2.36	16.3454	0.3844	43.06 ± 0.11 ^e^	47.99 ± 0.10 ^c^	56.28 ± 0.10 ^d^	3.91 ± 0.06 ^a^
glu/glo = 1:0 ^3^	0.6685 ± 0.0044 ^d^	5.74	−2.10	2.10	14.1705	0.4434	44.36 ± 0.23 ^d^	48.56 ± 0.16 ^b^	57.36 ± 0.21 ^c^	1.80 ± 0.06 ^d^
glu/glo = 2:1	0.7765 ± 0.0051 ^b^	8.09	−2.13	2.13	14.8363	0.4235	45.79 ± 0.10 ^c^	48.47 ± 0.08 ^b^	57.89 ± 0.07 ^b^	2.44 ± 0.04 ^c^
glu/glo = 1:1	0.7473 ± 0.0042 ^c^	7.09	−2.10	2.10	14.7251	0.4267	46.10 ± 0.18 ^c^	48.46 ± 0.14 ^b^	57.50 ± 0.20 ^c^	2.16 ± 0.04 ^c^
glu/glo = 1:2	0.7970 ± 0.0058 ^a^	9.85	−2.13	2.13	15.1768	0.4140	47.32 ± 0.08 ^b^	48.98 ± 0.07 ^a^	58.38 ± 0.11 ^a^	2.63 ± 0.05 ^b^
glu/glo = 0:1	0.8063 ± 0.0085 ^a^	7.19	−2.15	2.15	15.4113	0.4077	47.70 ± 0.16 ^a^	49.11 ± 0.11 ^a^	58.26 ± 0.11 ^a^	2.71 ± 0.04 ^b^

^1^ PRSG is the pure rice starch gel as a control. ^2^ Values followed by different letters within a column denote significantly different levels (*p* <0.05). ^3^ glu/glo is the glutelin-to-globulin ratio.

## Data Availability

The original contributions presented in the study are included in the article; further inquiries can be directed to the corresponding authors.
